# Androgen-Induced, β-Catenin-Activated Hepatocellular Adenomatosis with Spontaneous External Rupture

**DOI:** 10.3390/diagnostics14141473

**Published:** 2024-07-09

**Authors:** Jialing Huang, Towhid Ali, David M. Feldman, Neil D. Theise

**Affiliations:** 1 Department of Pathology, Geisinger Medical Center, Geisinger Commonwealth School of Medicine, 100 N. Academy Ave, Danville, PA 17822, USA; 2 Department of Radiology, Geisinger Medical Center, Geisinger Commonwealth School of Medicine, 100 N. Academy Ave, Danville, PA 17822, USA; 3 Department of Gastroenterology and Hepatology, New York University Grossman School of Medicine, 550 1st Avenue, New York, NY 10016, USA; 4 Department of Pathology, NYU Langone Medical Center, New York University Grossman School of Medicine, 550 1st Avenue, New York, NY 10016, USA

**Keywords:** hepatocellualr adenomatosis, β-catenin, androgen, liver rupture, EZH2

## Abstract

Androgens have long been recognized as oncogenic agents. They can induce both benign and malignant hepatocellular neoplasms, including hepatocellular adenoma (HCA) and hepatocellular carcinoma, though the underlying mechanisms remain unclear. Androgen-induced liver tumors are most often solitary and clinically silent. Herein, we reported an androgen-induced HCA complicated by spontaneous rupture. The patient was a 24-year-old male presenting with fatigue, diminished libido, radiology-diagnosed hepatocellular adenomatosis for 3 years, and sudden-onset, severe, sharp, constant abdominal pain for one day. He used Aveed (testosterone undecanoate injection) from age 17 and completely stopped one year before his presentation. A physical exam showed touch pain and voluntary guarding in the right upper quadrant of the abdomen. An abdominal CT angiogram demonstrated multiple probable HCAs, with active hemorrhage of the largest one (6.6 × 6.2 × 5.1 cm) accompanied by large-volume hemoperitoneum. After being stabilized by a massive transfusion protocol and interventional embolization, he underwent a percutaneous liver core biopsy. The biopsy specimen displayed atypical hepatocytes forming dense cords and pseudoglands. The lesional cells diffusely stained β-catenin in nuclei and glutamine synthetase in cytoplasm. Compared to normal hepatocytes from control tissue, the tumor cells were positive for nuclear AR (androgen receptor) expression but had no increased EZH2 (Enhancer of Zeste 2 Polycomb Repressive Complex 2 Subunit) protein expression. The case indicated that androgen-induced hepatocellular neoplasms should be included in the differential diagnosis of acute abdomen.

## 1. Introduction

Hepatocellular adenomas (HCAs) are benign liver tumors consisting of monoclonal neoplastic hepatocytes arranged in single-cell plates, with an incidence of 3/1,000,000 per year [1]. Four HCA subtypes are classified on both 2010 and 2019 WHO classifications: hepatocyte nuclear factor 1α inactivated HCA (H-HCA), inflammatory HCA (I-HCA), β-catenin-activated HCA (b-HCA), and unclassified HCA (u-HCA) [2,3], though other rarer subtypes have now been identified, such as sonic hedgehog activation-HCA(sh-HCA) [4] and myxoid HCA [5]. H-HCA and I-HCA are the most common subtypes. Immunohistochemical staining with appropriate antibodies can aid in the correct diagnosis. 

HCAs occur most commonly in women of reproductive age. Several etiologies can cause them: (1) estrogen, including use of oral contraceptives (OCPs) [6]; (2) anabolic androgen steroids [7]; and (3) abnormal carbohydrate metabolism, including glycogen storage disease type I and III, obesity, glucose intolerance, and diabetes, especially maturity-onset diabetes of the young type 3 (MODY3) [8,9]. It is known that long-term use of OCPs is strongly linked to the development of HCAs; for example, 88% of cases of hepatocyte nuclear factor 1alpha (*HNF1A*)-mutated HCA (H-HCA) and 77% of cases of I-HCA are women taking OCPs [8]. Accumulating data indicate that estrogen plays a multi-faceted role in the pathogenesis of HCAs. First, catechol estrogens can be oxidized to quinone, which mediates the formation of DNA adducts, leading to direct DNA damage and causing inactivating mutations of *HNF1A* [10]. This can explain the role of OCPs in the development of H-HCA. Second, it is speculated that estrogen promotes I-HCA by directly activating the IL-6/JAK/STAT/p130 pathway [8,11]. Another contributory genetic factor for the formation of H-HCA in OCP-taking women is mutations of the *CYP1B1* gene [12]. However, the mechanisms underlying androgen-induced HCAs have not been elucidated yet.

HCAs carry two major risks. First, they are commonly complicated by hemorrhage/rupture due to hormone-dependent tumor growth, sinusoidal dilatation, and the lack of a capsule. The rate of hemorrhage and rupture is reportedly up to 52%, and the symptomatic hemorrhage rate is 14% [13]. Particularly, special attention should be given to pregnant women with HCA, as tumor rupture is life-threatening for both mother and fetus [14,15]. Among the molecular subtypes, b-HCA with mutations in *CTNNB1* exons 7 and 8 has the highest hemorrhage rate of 92%, followed by sh-HCA, which is associated with hemorrhage in 81% of the cases, while hemorrhage risk in b-HCAs with mutations in *CTNNB1* exon 3 is 69%. Specifically, most hemorrhages in b-HCAs are internal, resulting from rupture inside the tumor, and for this reason, intraperitoneal hemorrhage is relatively rare. Second, HCAs harbor a significant risk of malignant transformation. The highest risk of this transformation is due to mutations in the *TERT* promoter. Other risk factors are, in descending order, male gender, *CTNNB1* exon 3 mutations, unique nodules at imaging, high alcohol intake, fibrosis in the non-tumor liver, and diabetes type 2 [8]. 

It is well documented that androgen overstimulation can cause hepatocellular adenoma, liver adenomatosis, and hepatocellular carcinoma [16,17]. This association was first brought to attention in patients taking exogenous androgens for hematologic diseases such as Fanconi anemia (FA) [18,19]. It is not surprising that the association has also been seen in bodybuilders, gender identity disorder, conditions with overproduction of endogenous androgens such as polycystic ovary syndrome, and androgen therapy for non-FA anemia [20,21,22]. Androgen-induced HCA most commonly occurs in males and is often β-catenin activated due to somatic mutations in *CTNNB1* exon 3 [16]. So far, more than 10 cases of anabolic androgen-induced HCA without FA have been reported [16,21,23,24,25,26,27,28]. Most of the patients had multiple lesions. Although internal hemorrhage was seen in one patient [28], none was complicated by intraperitoneal rupture. 

Herein, we report that a 24-year-old male bodybuilder presented with massive hemoperitoneum resulting from intraperitoneal rupture of hepatocellular adenomatosis. The tumor was diagnosed as b-HCA by standard immunostaining assays, which also showed expression of AR (androgen receptor) in the lesional cells, indicating receptor activation, but with no increase in EZH2 (Enhancer of Zeste 2 Polycomb Repressive Complex 2 Subunit) protein expression. 

## 2. Case Presentation 

A 24-year-old male, radiographically (contrast-enhanced abdominal CT) diagnosed with hepatocellular adenomatosis 3 years previously, now presented with fatigue, diminished libido, and sudden-onset, severe, sharp, constant abdominal pain for one day. The pain was localized to the right upper quadrant, non-radiating, and associated with chills, but not accompanied by fever, nausea, or vomiting. His past medical history was significant for asthma, use of Aveed (testosterone undecanoate injection) from age 17, and secondary hypogonadism likely due to anabolic steroid use. Androgen administration was completely withdrawn one year prior to presentation, but there were no changes in tumor number or size on radiological studies at a 6-month follow-up. Physical exam: non-obese, body mass index (BMI) 20, body temperature 37 °C, heart rate 130, respiratory rate 28, blood pressure 98/59 mmHg, abdomen with touch pain, and voluntary guarding in the right upper quadrant. 

Laboratory results showed the following: white blood cell count 11.5 (normal 4–10.8 K/uL), platelet 433 (normal 140–400 K/uL), aspartate aminotransferase 147 (AST, normal 10–35 U/L), alanine transaminase 389 (ALT, normal 10–35 U/L), lipase 105 (normal 0–160 U/L), C-reactive protein 114.6 (normal 0–5 mg/L), alpha-fetoprotein < 2 (AFP, normal < 8.8 ng/mL), total protein 5.2 (normal 6.7–8.6 g/dL), albumin 3.0 (normal 3.5–5.2 g/dL), and alkaline phosphatase 34 (ALP, normal 40–150 U/L). Endocrine lab data: testosterone > 1500 (normal 300–1000 ng/dL), follicle-stimulating hormone < 0.7 (FSH, normal adult male 1.5–12.4 mIU/mL), luteinizing hormone < 0.2 (LH, normal adult male 1.7–8.6 mIU/mL), estradiol 365 (normal 14–43 pg/mL), prolactin 37.2 (normal 5–18 ng/mL), and 5(OH)D 18.7 (normal 20–40 ng/mL). 

**Figure 1:**
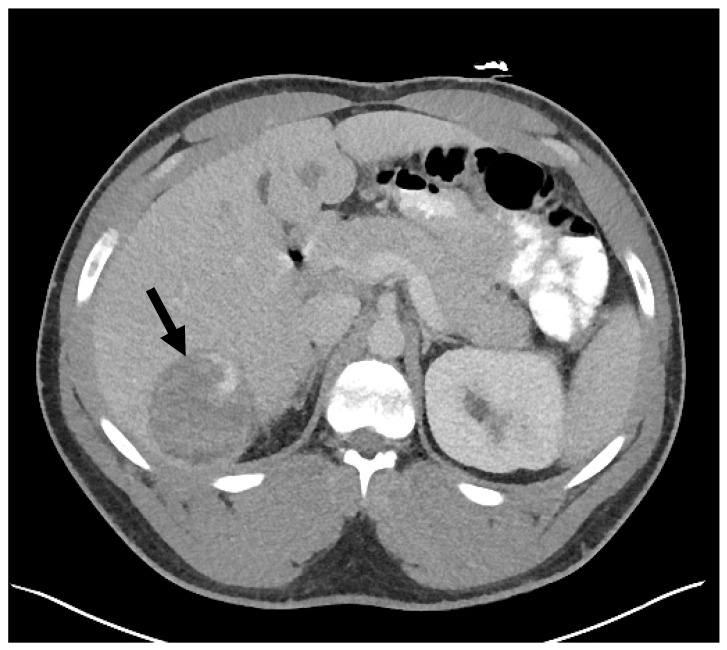
Axial abdominal contrast-enhanced CTT showing the largest hepatocellular adenoma that ruptured with active bleeding.

Although bedside FAST (Focused Assessment with Sonography in Trauma) examination was negative, abdominal CT angiography demonstrated: (1) an internally ruptured 6.6 × 6.2 × 5.1 cm heterogenous lesion in segment 6, most likely representing hepatocellular adenoma, with a blush of contrast within the lesion which pooled on the delayed venous phase, indicating active hemorrhage into the lesion (Figure 1, arrow); (2) multiple additional hypervascular lesions with washout within segments 3, 4B, and 5 measuring up to 4.3 × 5.4 cm in segment 5, likely also representing hepatocellular adenomas; and (3) a large-volume abdominal and pelvic hemoperitoneum. 

The patient underwent a massive transfusion protocol and interventional embolization of subsegmental hepatic artery branches in segment 6 with embospheres. A simultaneous drainage of 1.5 L of intraperitoneal blood was also carried out. The patient was complicated by post-operative small bowel obstruction 10 days later, which was successfully managed with laparotomy for lysis of adhesions. A percutaneous core biopsy was performed to sample the lesions in segments 3 and 4B.

## 3. Pathologic Findings 

**Figure 2:**
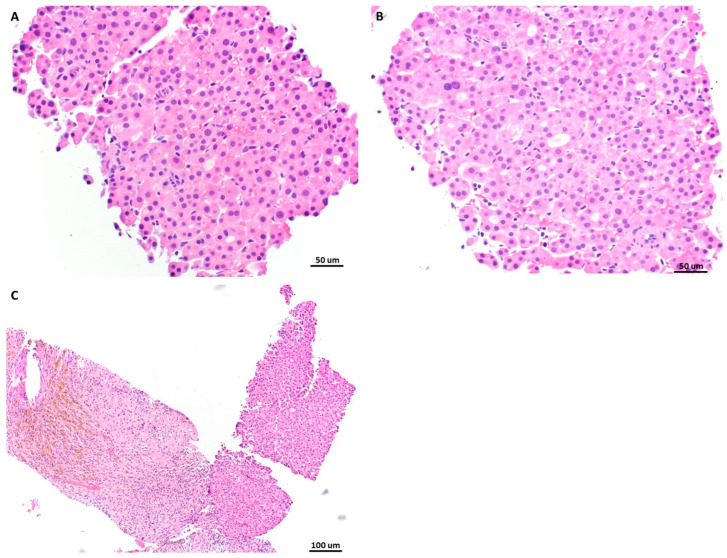
Histologic changes of the lesions on H&E stain. Lesions on segments 3 (**A**) and 4B (**B**) were both composed of uniform hepatocytes with mild cytologic atypia, growing in dense cords with occasional foci of pseudoglandular architecture. Hemosiderin, giant cell reaction, hyalinization, and necrosis in tissue from segment 4B suggested prior embolization therapy (**C**).

Lesional tissue on the biopsy cores was comprised of mature and uniform-appearing hepatocytes with mild cytologic atypia, growing in dense cords with occasional foci of pseudogland formation (Figure 2A,B). Evidence of hemosiderin, giant cell reaction, hyalinization, and necrosis in tissue from segment 4B, consistent with status post-embolization therapy, was also seen (Figure 2C). 

**Figure 3:**
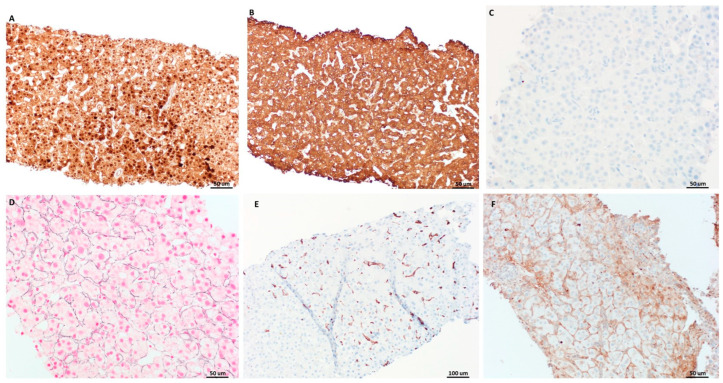
The lesional tissue from segments 3 and 4B shared similar immunophenotypes. The β-catenin immunostains showed diffuse nuclear staining in lesional cells (**A**), while glutamine synthetase was strongly and diffusely positive (**B**). Lesional cells were stained negative for glypican-3 (**C**). Although reticulin stains highlighted patchy foci of disruption and loss (**D**), CD34 immunostain displayed a patchy sinusoidal pattern of staining (**E**). C-reactive protein immunostains were negative (**F**).

The lesional tissue from segments 3 and 4B also shared similar immunophenotype: β-catenin immunostains (Ventana Medical Systems, Santa Clara, CA, USA, catalog # 760-4242) showed diffuse nuclear staining, glutamine synthetase (Ventana Medical Systems, Santa Clara, CA, USA, catalog # 760-4898) was strongly and diffusely positive, and glypican-3 (Roche, Indianapolis, IN, USA, catalog # 05973864001) was negative in lesional cells, compatible with the *CTNNB1* non-S45 exon 3 mutation pattern (Figure 3A–C) [29]. The simultaneous diffuse and strong expression of both β-catenin and glutamine synthetase indicated β-catenin activation in the lesional cells. Reticulin stains in both parts highlighted a predominantly preserved reticulin network with patchy foci of disruption and loss (Figure 3D). CD34 immunostains (Roche, Indianapolis, IN, USA, catalog # 05278210001) displayed a patchy sinusoidal pattern of staining in both parts, while C-reactive protein immunostains (Abcam, Waltham, MA, USA, catalog # Ab32412) were negative (Figure 3E,F).

The overall morphologic and immunophenotypic features favor the diagnosis of b-HCA in both parts. However, given the inherent sampling error associated with a biopsy, a more advanced unsampled lesion (hepatocellular carcinoma) could not be excluded based on the current specimens.

**Figure 4:**
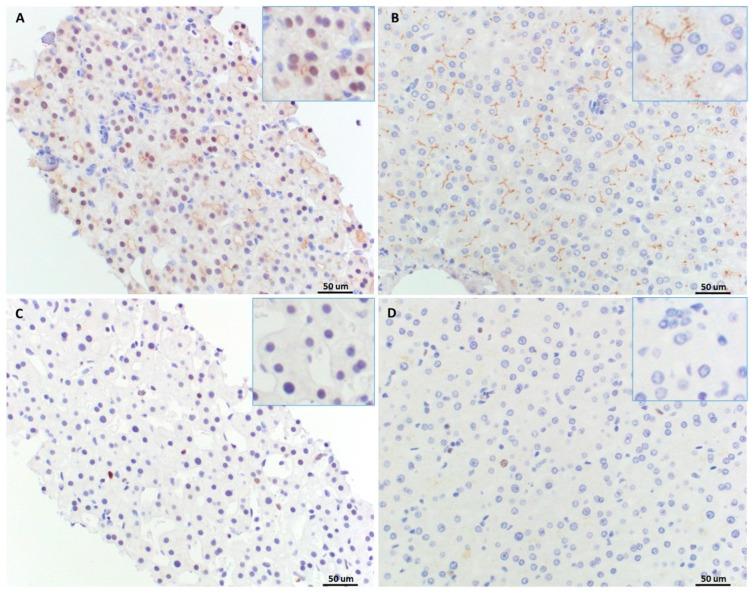
Androgen pathway activation in the lesions. AR and EZH2 expression was examined with immunohistochemistry in the lesions ((**A**) AR, (**C**) EZH2, respectively) and normal liver tissue ((**B**) AR, (**D**) EZH2, respectively). The rare positive signal was distributed in sinusoidal cells in (**C**). Insets for higher resolution of nuclear staining.

To investigate androgen pathway activation in this case, we assessed the activation of the AR-EZH2 (enhancer of zeste homolog 2) signal pathway by evaluating the expression of AR and EZH2 proteins immunohistochemically with two repeat experiments. The results consistently showed that the lesional cells had scattered nuclear expression of AR (Roche, catalog # 06523838001) with moderate staining intensity (Figure 4A). No nuclear staining of AR was revealed in the normal control hepatocytes from the non-lesional tissue of a resected liver specimen harboring focal nodular hyperplasia (Figure 4B). These results indicated specific activation of the AR signal pathway in this patient’s tumor. In contrast to the observed differential expression of AR in the tumor and normal liver tissue, no difference in EZH2 expression (ThermoFisher, Waltham, MA, USA, catalog # TA803011) was noticed—both the HCA and the normal liver tissue displayed rare, faintly stained cells (Figure 4C,D)—the staining displayed likely represents a background signal. Cananlicular staining of AR was noted in both HCA and normal liver tissue (Figures 4A and 4B, respectively). 

## 4. Discussion

The involvement of androgen in the carcinogenesis of liver tumors is evidenced by multiple lines of observation: First, extra exogenous or endogenous androgen is associated with the development of hepatocellular neoplasms, including both benign and malignant tumors [30,31]. Second, the prevalence of hepatocellular carcinoma is higher in males than females globally [32,33]. Lastly, androgen initiates carcinogenesis in hepatitis B virus-related HCC [34,35], likely by enhancing telomerase reverse transcriptase gene (*TERT*) expression [36]. In our case, the upregulated nuclear expression of AR in the lesional cells indicated activation of the AR signal pathway due to the administration of anabolic steroids. To our knowledge, this is the first direct observation of AR-EZH2 pathway signaling in androgen-driven HCA.

Although available clinical data consistently indicate an ominous role of AR in the development of HCC, the results of AR expression in human lesional tissues are limited and conflicting. In the early observations, AR was overexpressed in 60–80% of human HCCs, not only in the tumor but also in the peritumor liver tissue [37]. Of note, HCC tumor cells also express the estrogen receptor (ER). In addition, both androgen and estrogen receptors expressed in the neoplastic cells affect intrahepatic recurrence and recurrence-free 5-year survival [38]. Further study demonstrates that AR is usually expressed in HCC tumors smaller than 3 cm in diameter but not in later stages, and this temporal pattern of AR expression is not associated with postoperative survival. In contrast, AR expression level in the peritumoral parenchyma is associated with prognosis [39]. Similarly, ER and progestin receptors are expressed in both HCA and adjacent liver parenchyma as well [40]. Furthermore, anti-androgen therapy fails to show efficacy in unresectable HCC. These results imply that androgen may promote HCC in the initiation stage but not in the progression stage, which could explain the failure of therapy targeting the androgen pathway. In line with this point, just like in our case, most androgen-induced HCAs do not regress after hormone withdrawal [41,42,43]. Moreover, a recent study shows that AR expressed in the liver inhibits HCC metastasis [44]. Further complicating this topic, androgen pathways in the liver can also be activated by factors other than androgen, including, but not limited to, Her-2 and β-catenin [45,46]. The real role of hepatocytic androgen in hepatic carcinogenesis indeed needs further investigation. 

Androgen-driven hepatocellular neoplasms commonly display architectural and cytologic atypia and are therefore called well-differentiated hepatocellular neoplasms of uncertain malignant potential (HUMP) by some authors [16]. These tumors can harbor *CTNNB1* mutations in exon 3 or 7, which result in self-phosphorylation of β-catenin, leading to activation of the Wnt pathway [16,23,47]. Inflammatory and other pathways are also possibly involved. Oral contraceptives were speculated 40 years ago to be able to upregulate the expression of estrogen and androgen receptors in the liver [48]. Although immunohistochemical assessment of AR is seen in scattered literature [31], the association between androgen use and AR expression is still not well established, as is the relationship between androgen–AR signaling and β-catenin activation [49]. In fact, there is still no widely accepted cutoff for AR and β-catenin immunohistochemical positivity in liver pathology so far. So, there is no wonder why the reported AR and β-catenin positivity in liver tumors is remarkably varied among the limited literature, making direct comparison of the results difficult and assessment of the dose effect of androgen on hepatocellular oncogenesis impossible. In our case, immunohistochemical studies demonstrated diffuse and homogeneously intense nuclear staining of β-catenin, in addition to disproportional, scattered AR expression in the lesional cells. The results imply an amplification of the action of androgen in the androgen–β-catenin cascade in the tumor cells. Because molecular-classified non-b-HCAs can contain some tumor cells with positive nuclear staining of β-catenin [50], we cannot exclude the possible role of other β-catenin inducers in the malignant transformation of the tumor cells in our case. We believe that molecular profiling will help provide valuable information on the molecular pathway(s) involved in this process. 

Nevertheless, recent studies have shown that androgen–AR signaling may trigger two pathways mediating activation of β-catenin in hepatocellular carcinogenesis. The first one is led by the CCRK4 protein (cell cycle-related kinase 4, also known as cyclin-dependent kinase 20), which can serve as an oncogenic effector of AR in HCC [51]. The second one is conveyed by the EZH2 protein (enhancer of zeste homolog 2), whose expression is inducible upon AR signaling [52]. Intercommunication between these two pathways is also implied in hepatocarcinogenesis [53]. In our case, the increased AR expression in the absence of upregulation of EZH2 protein in the HCA cells suggests activation of the β-catenin pathway by androgen–AR signaling via a mechanism mediated by molecules other than EZH2. Exploration of the CCRK4 pathway was not attempted due to limited research resources. It is known that AR signaling can regulate the interaction between HBx and CCRK molecules, leading to HBV (hepatitis B virus) carcinogenesis [54]. The exact AR signaling cascade leading to β-catenin activation in our case needs further investigation.

Another unusual observation in our study is the canalicular pattern of AR staining in both the lesional and normal liver tissue, repeatedly shown in separate experiments. This pattern has not been reported elsewhere, and we have no explanation for it as yet. The nature of the antibody or the antigen used to generate the antibody might contribute to it, perhaps with a specific cross-reaction to a canalicular protein, as is seen, for example, with polyclonal anti-CEA antibodies [55].

## 5. Conclusions

In summary, we reported a case of androgen/AR signaling-induced hepatocellular adenomatosis complicated by spontaneous external rupture. This condition should be considered in patients with acute abdomen, especially those with androgen exposure. 

## Data Availability

The data presented in this study are available on request from the corresponding author. The data are not publicly available due to being clinical data.
